# Fatty Acid-Rich Fraction of *Hibiscus syriacus* L. Alleviates Atopic Dermatitis-like Skin Lesions Mouse Model via Inflammatory Pathway Modulation: Integrative Docking and Experimental Validation

**DOI:** 10.3390/plants14152447

**Published:** 2025-08-07

**Authors:** Trang Thi Minh Nguyen, Bom Park, Xiangji Jin, Qiwen Zheng, Gyeong-Seon Yi, Su-Jin Yang, Tae-Hoo Yi

**Affiliations:** 1 Graduate School of Biotechnology, Kyung Hee University, 1732, Yongin-si 17104, Republic of Korea; trangnguyen@khu.ac.kr (T.T.M.N.); viesna91@khu.ac.kr (B.P.); zhengqiwen@khu.ac.kr (Q.Z.); stella@khu.ac.kr (S.-J.Y.); 2 Department of Dermatology, School of Medicine, Kyung Hee University, 23 Kyungheedae-ro, Dong-daemun, Seoul 02447, Republic of Korea; hyanghe112@khu.ac.kr; 3 Department of Biopharmaceutical Biotechnology, Graduate School, Kyung Hee University, 1732 Deogyeong-daero, Giheung-gu, Yongin-si 17104, Republic of Korea; ks010924@khu.ac.kr

**Keywords:** atopic dermatitis, corticosteroid, fatty acids, *Hibiscus syriacus*, inflammation, molecular docking

## Abstract

Atopic dermatitis (AD) remains a therapeutic challenge due to the limitations of current treatments, creating demand for safer multi-target alternatives to corticosteroids. Our integrated study establishes *Hibiscus syriacus* L. (*H. syriacus*) as a mechanistically validated solution through computational and biological validation. The fraction’s two main compounds, linoleic acid and palmitic acid, exhibit favorable drug-like properties including high lipophilicity (LogP 5.2) and 87% oral absorption. Molecular docking collectively predicts comprehensive NF-κB pathway blockade. Experimental validation showed that the fraction (100 μg/mL) inhibited LPS-induced nitric oxide (NO) by 78% and TNF-α/IFN-γ-induced reactive oxygen species (ROS) by 40%, while significantly downregulating the chemokines TARC (73%) and MDC (71%). In DNCB-induced AD mice, the treatment (200 mg/kg/day) produced a 62% improvement in clinical severity scores, reduced serum IgE by 27%, decreased transepidermal water loss by 36%, and doubled skin hydration while normalizing pH levels from the alkaline to physiological range. While both treatments reduced DNCB-induced epidermal hyperplasia, *H. syriacus* (62.9% reduction) restored the normal thickness without pathological thinning, a critical advantage over corticosteroids that cause atrophy. This dual-action therapeutic achieves corticosteroid-level anti-inflammatory effects while restoring skin barrier integrity to normal levels and avoiding corticosteroid-associated atrophy, positioning it as a next-generation AD treatment.

## 1. Introduction

Atopic dermatitis (AD) affects 15–25% of children and 2–10% of adults worldwide, with approximately 170 million cases, with its rising prevalence linked to climate change and pollution [1,2]. This chronic inflammatory disease involves a destructive cycle of skin barrier dysfunction and immune dysregulation, driven by Th2-mediated IgE overproduction [3,4]. Current treatments such as tacrolimus (immunosuppressant) and prednisolone (corticosteroid) suppress symptoms but carry risks such as immunosuppression, skin atrophy, and systemic effects, including renal dysfunction and increased cancer risk with prolonged use [5]. Compounding these challenges, 20–80% of AD patients develop food allergies, underscoring the need for safer alternatives [6]. The disease’s complexity, involving NF-κB/MAPK pathways, Th1/Th2 imbalance, and ROS-mediated damage, demands multi-target approaches [7]. Natural products offer unique potential by simultaneously addressing barrier repair [8] and immune modulation while reducing key chemokines such as TARC, MDC, and RANTES [9]. This comprehensive strategy represents a critical advance over current single-target therapies, particularly given AD’s environmental triggers and systemic nature.

*Hibiscus syriacus* L. (*H. syriacus*), a traditional medicinal plant native to Asia and revered as Korea’s national flower (“Mugunghwa”) [10], has emerged as a compelling candidate for atopic dermatitis (AD) therapy. Phytochemical analyses of *H. syriacus* reveal a rich profile of bioactive compounds, including linoleic acid (13.11%) and palmitic acid (5.9%) [11], fatty acids critical for skin barrier repair and possessing notable antioxidant activity [12,13]. Notably, its root extract exhibits anti-photoaging effects via MMP and filaggrin modulation [14], while its flower extracts accelerate cutaneous repair in vivo [15]. While *H. syriacus* has a traditional use profile that supports its relevance to skin inflammation [16] and modern preclinical studies suggest anti-inflammatory and barrier-enhancing properties [17,18], direct ethnomedical or clinical studies on its effect on AD specifically are still limited [10]. Unlike current AD treatments, *H. syriacus* offers a multitarget approach, immune dysregulation (Th2 cytokine inhibition), and barrier dysfunction, without systemic toxicity risks. With centuries of ethnomedical use and recent validation in dermatological models, *H. syriacus* presents a clinically translatable, natural alternative to conventional immunosuppressants [10].

Given the current side effects of atopic dermatitis treatments, including skin atrophy, purpura, telangiectasia, and facial acneiform changes [5], this study aims to evaluate the therapeutic potential of *H. syriacus* in managing AD. We focus on the fatty acid-rich fraction of *H. syriacus* to assess its ability to alleviate AD-like symptoms through computational modeling, TNF-α/IFN-γ-induced cell assays, and DNCB (2,4-dinitrochlorobenzene)-induced mouse models, targeting both inflammation and skin barrier dysfunction. This work seeks to provide mechanistic evidence for *H. syriacus* as a novel, multi-target botanical therapy for AD, addressing both inflammation and barrier repair while minimizing side effects commonly associated with conventional treatments.

## 2. Results

### 2.1. Identification of Major Compounds and Molecular Docking Analysis of Hibiscus syriacus Fatty Acid-Rich Fraction

**Figure 1:**
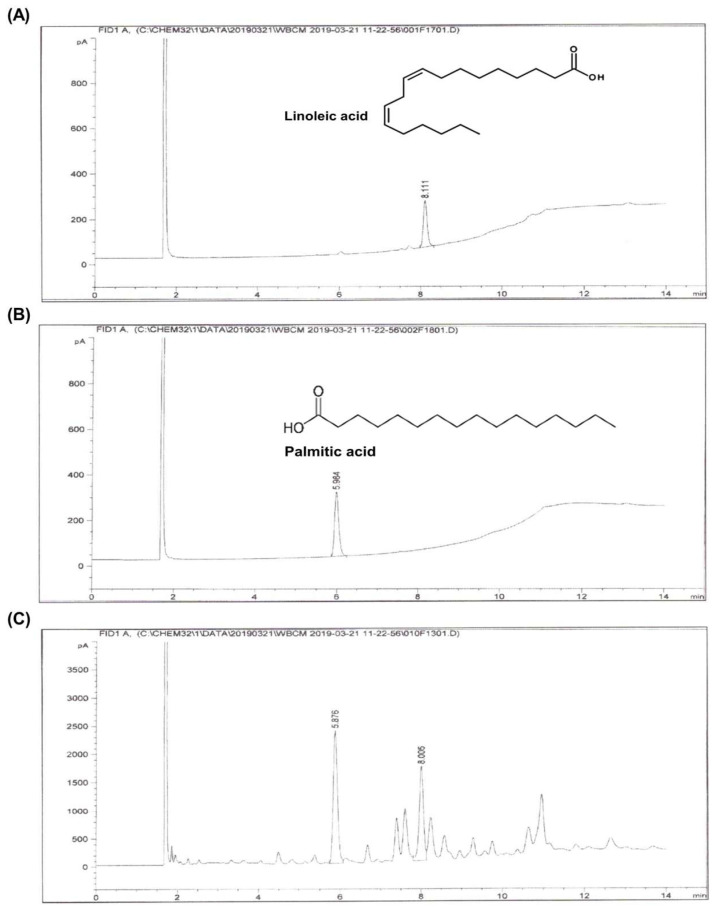
A GC-MS analysis of *Hibiscus syriacus* fatty acid-rich fraction. Representative gas chromatogram of *H. syriacus* fatty acid-rich fraction, identifying total linoleic acid (**A**) and palmitic acid (**B**) as the major bioactive components, and the full chromatogram of *H. syriacus* fatty acid-rich fraction (**C**).

A GC-MS analysis of the *Hibiscus syriacus* fatty acid-rich fraction (yield at 1.23% w/w) identified total linoleic acid (10.3%) and palmitic acid (14.1%) (free and esterified forms) as the major constituents (Figure 1). A QikProp analysis showed that both compounds exhibited high lipophilicity (LogP: 5.291 for linoleic acid; 5.251 for palmitic acid) and high human oral absorption (87.5% and 87.2%, respectively). Each compound had one hydrogen bond donor and two hydrogen bond acceptors, with moderate dipole moments (6.52 and 6.83 Debye). Linoleic acid had a polar surface area of 37.5 Å^2^, whereas palmitic acid showed 0.0 Å^2^. Both were predicted to be inactive in the CNS (score: –2) and had good permeability across Caco-2 cell monolayers (239.37 nm/s and 235.46 nm/s). Each compound violated one of Lipinski’s rules due to the LogP values slightly exceeding the threshold. Detailed ADME properties are summarized in Table 1.

**Figure 2:**
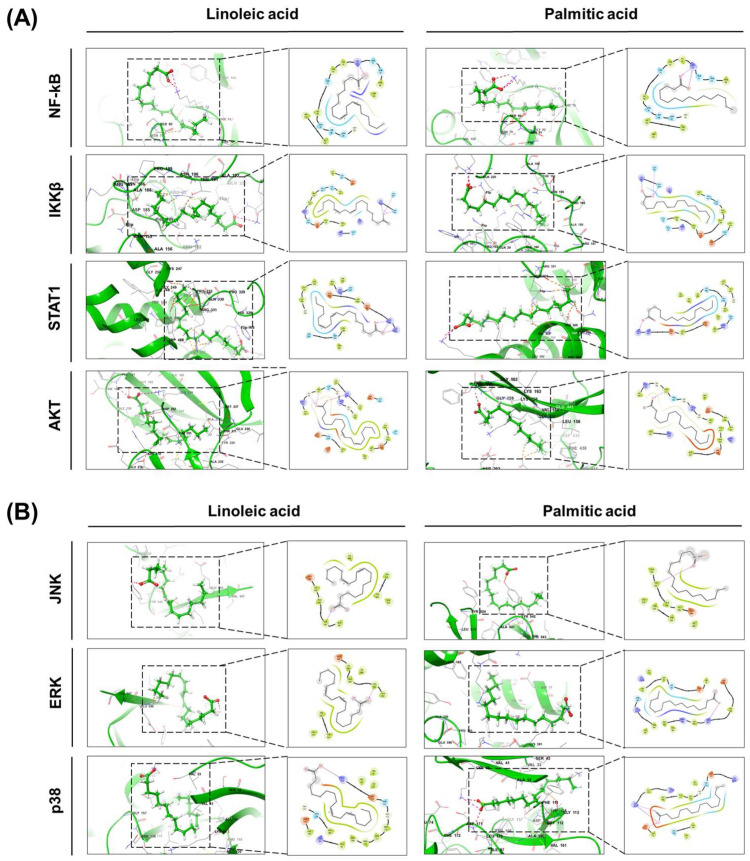
A molecular docking analysis of the two main components of *Hibiscus syriacus*, linoleic acid and palmitic acid, with key inflammatory signaling proteins: (**A**) binding interactions with canonical inflammatory pathway targets (NF-κB, IKKβ, STAT1, AKT); (**B**) binding interactions with MAPK signaling pathway proteins (JNK, ERK, p38).

Molecular docking was performed using the Glide XP module with OPLS4 force field. Linoleic acid showed the strongest binding to AKT (Emodel: –35.18), followed by p38 (–30.55), NF-κB (–26.66), and ERK (–26.67). Palmitic acid demonstrated the highest affinity for IKKβ (–22.78), followed by p38 (–24.71) and AKT (–22.42). Both compounds showed lower binding energies with JNK and STAT1. The complete docking results are listed in Figure 2 and Table 2.

### 2.2. Effects of Hibiscus Syriacus Fatty Acid-Rich Fraction on NO and ROS Production

**Figure 3:**
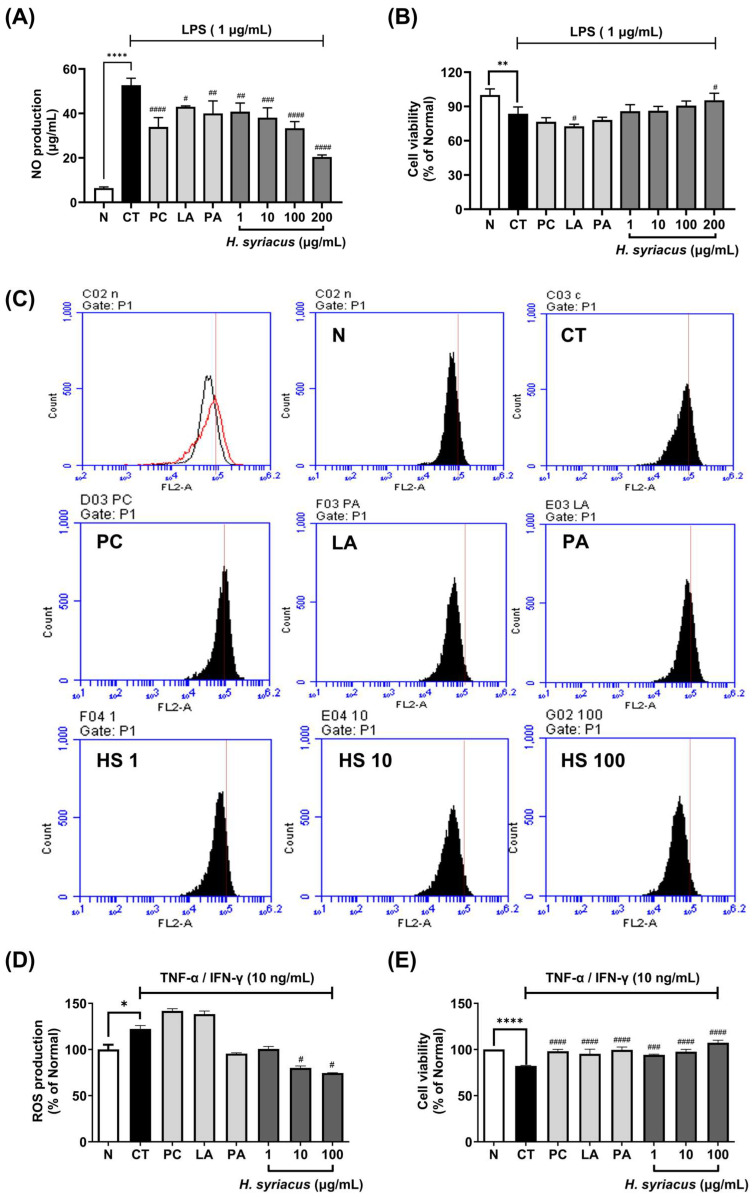
Effects of *Hibiscus syriacus* fatty acid-rich fraction on nitric oxide (NO) and reactive oxygen species (ROS) production in vitro. NO production (**A**) and cell viability of 1 µg/mL LPS-stimulated RAW 264.7 macrophages treated with *H. syriacus* (**B**). Intracellular ROS production in TNF-α/IFN-γ-stimulated HaCaT cells at 10 ng/mL (**C**,**D**) and its effect on cell viability (**E**). LA (linoleic acid, 1 µg/mL), PA (palmitic acid, 1 µg/mL), and tacrolimus (10 µg/mL) were used as the positive controls. Data are presented as the mean ± SEM. Statistical significance is indicated as * *p <* 0.05, ** *p <* 0.01, **** *p <* 0.0001 vs. normal group, # *p <* 0.05, ## *p <* 0.01, ### *p <* 0.001, #### *p <* 0.0001 vs. control group.

The cytotoxicity assay confirmed that the *Hibiscus syriacus* fatty acid-rich fraction exhibited no adverse effects on the RAW 264.7 macrophage viability at the tested concentrations. Upon LPS stimulation, the NO levels increased dramatically by 821.5% compared to the unstimulated control. Treatment with *H. syriacus* (100 µg/mL) significantly suppressed LPS-induced NO production by 78.03% (*p <* 0.0001) without compromising the cell viability (Figure 3A,B). Among the major constituents, linoleic acid and palmitic acid also reduced the NO generation significantly, with 23.07% and 18.58% inhibition rates, respectively, at lower concentrations.

In HaCaT keratinocytes, TNF-α/IFN-γ co-stimulation induced substantial intracellular reactive oxygen species (ROS) production. *H. syriacus* treatment significantly attenuated ROS accumulation, showing effects comparable to those of dexamethasone (*p <* 0.05, Figure 3B). A fluorescence analysis further revealed dose-dependent ROS reduction rates of 18.03%, 34.43%, and 39.35% at 1, 10, and 100 µg/mL of the extract, respectively (Figure 3C–E). Among the isolated compounds, palmitic acid decreased ROS levels by 22.13%, while linoleic acid did not show a significant reduction under these conditions. Interestingly, treatment with tacrolimus slightly increased ROS production in this model (Figure 3C–E).

### 2.3. Hibiscus syriacus Fatty Acid-Rich Fraction Suppresses Pro-Inflammatory Cytokine and Chemokine Expression

**Figure 4:**
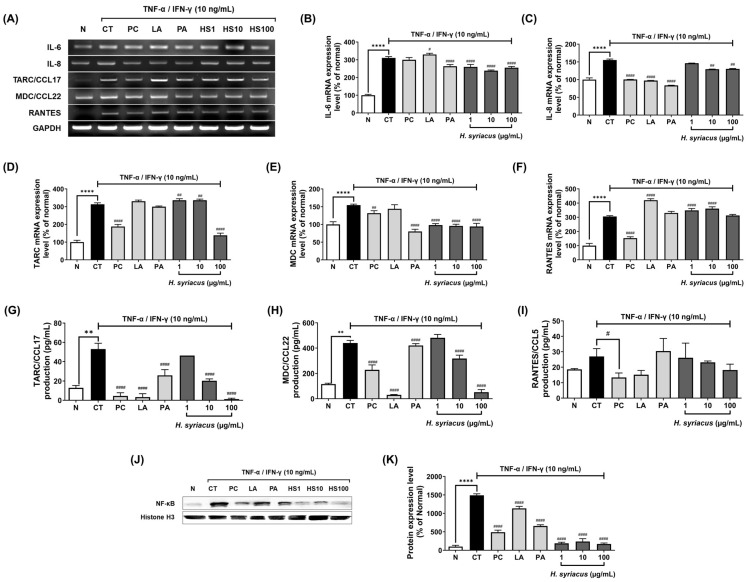
Suppression of cytokine expression by Hibiscus syriacus fatty acid-rich fraction in TNF-α/IFN-γ-stimulated HaCaT cells: (**A**–**F**) mRNA expression of IL-6, IL-8, TARC, MDC, and RANTES; (**G**–**I**) production levels of TARC, MDC, and RANTES via ELISA; (**J**,**K**) protein expression of NF-κB in Western blot. LA (linoleic acid, 1 µg/mL), PA (palmitic acid, 1 µg/mL), and tacrolimus (10 µg/mL) were used as the positive controls. Data are presented as the mean ± SEM. Statistical significance is indicated as ** *p* < 0.01, **** *p* < 0.0001 vs. normal group, # *p* < 0.05, ## *p* < 0.01, #### *p* < 0.0001 vs. control group.

To investigate whether the *H. syriacus* fatty acid-rich fraction suppresses the mRNA expression of pro-inflammatory mediators, HaCaT cells were pretreated with *H. syriacus* or tacrolimus for 30 min, followed by stimulation with TNF-α and IFN-γ for 6 h. Based on prior cytotoxicity results, RT-PCR was conducted at non-toxic concentrations (1–100 µg/mL). Following TNF-α/IFN-γ stimulation, there was a marked increase in mRNA levels of IL-6 (310.5%), IL-8 (155.3%), TARC/CCL17 (313.4%), MDC/CCL22 (154.4%), and RANTES/CCL5 (305.4%) compared to unstimulated controls (Figure 4). Pretreatment with *H. syriacus* significantly reduced the overexpression of IL-6, TARC/CCL17, and MDC/CCL22, with respective decreases of 85.1%, 73.6%, and 71.4%. Also observed with tacrolimus were reductions in IL-6 (96.6%), IL-8 (64.6%), TARC/CCL17 (60.0%), MDC/CCL22 (85.4%), and RANTES (50.1%). However, compared to the TNF-α/IFN-γ group, tacrolimus slightly increased IL-6 and RANTES levels, suggesting partial rebound activation. Interestingly, palmitic acid treatment led to a greater suppression of IL-6 (87.9%) and MDC/CCL22 (60.7%) mRNA expression than the positive control (tacrolimus at 10 µg/mL) (Figure 4).

**Figure 5:**
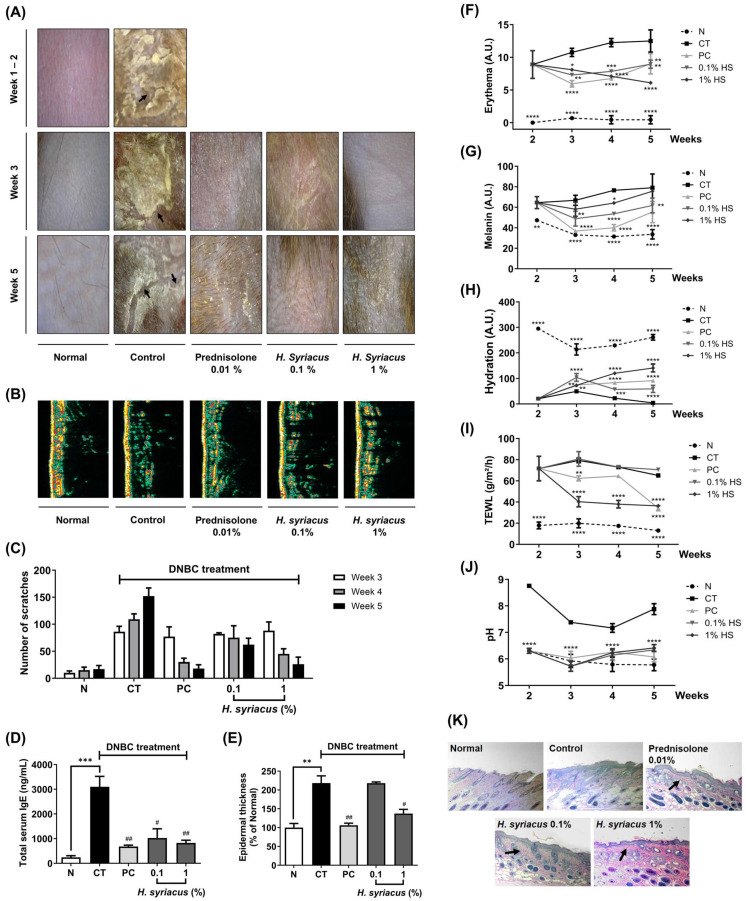
Therapeutic effects of *Hibiscus syriacus* fatty acid-rich fraction on DNCB-induced atopic dermatitis in NC/Nga mice in 5 week treatment: (**A**) improvement in physiological alterations; (**B**) reduction in epidermal skin density; (**C**) suppression of scratching behavior; (**D**) decrease in serum IgE levels; (**E**) attenuation of epidermal skin thickness; (**F**–**J**) modulation of skin surface parameters, including erythema (**F**), melanin (**G**), hydration (**H**), transepidermal water loss (TEWL) (**I**), pH (**J**), and photomicrographs (200×) of H&E stained sections (**K**). N represents the normal group, CT indicates the DNCB-treated control group, and PC denotes the positive control group treated with 0.01% prednisolone. Data are presented as the mean ± SD; * *p <* 0.05, ** *p <* 0.01, *** *p <* 0.001, **** *p <* 0.0001 vs. normal group; # *p <* 0.05, ## *p <* 0.01 vs. control group.

The therapeutic effects of the *Hibiscus syriacus* fatty acid-rich fraction were evaluated on DNCB-induced AD in NC/Nga mice over a 5-week period, assessing various physiological and histological parameters. During the study, there were no significant differences in body weight across all groups, including the normal, DNCB-treated control (CT), positive control (PC) treated with prednisolone 0.01%, and *H. syriacus*-treated groups. The skin lesions caused by DNCB, such as erythema, edema, and crusting, progressively worsened. However, treatment with *H. syriacus* and prednisolone alleviated these skin alterations, as confirmed by photographs using the Dermalab skin measurement system (Figure 5A). Ultrasonic measurements of skin density revealed significant impairment in the DNCB-treated group. However, treatment with either prednisolone or *H. syriacus* improved the skin density. This restoration was visually confirmed through DermaLab Combo system 2.2.7.1 (Cortex Technology, Denmark), showing green–yellow color patterns indicating a recovery in skin density (Figure 5B).

The DNCB-treated mice exhibited intense scratching behavior due to pruritus. A video analysis showed that the frequency of scratching was significantly reduced in the prednisolone and 1% *H. syriacus* groups throughout the experiment (Figure 5C). Serum IgE levels were significantly elevated (1275.5%) in the DNCB group compared to the normal group. Both prednisolone and *H. syriacus* treatments significantly reduced serum IgE levels, with prednisolone reducing the levels by 21.91% and *H. syriacus* by 26.6% (Figure 5D).

The epidermal thickness in the DNCB-treated mice increased by 218.2%. In contrast, treatment with prednisolone and *H. syriacus* resulted in significant decreases in epidermal thickness by 48.6% and 62.9%, respectively, with the *H. syriacus* effect being 1.5-fold higher than prednisolone (Figure 5E,K).

The DNCB treatment significantly increased the erythema index. However, the erythema gradually decreased with treatment, with *H. syriacus* showing a continuous improvement in erythema, while prednisolone had a faster response in the early stages of treatment, which decreased in effect over weeks 3 to 5 (Figure 5F). At week 5, *H. syriacus* reduced the erythema to 50% of the control group, while prednisolone (PC) reduced it to 75% of the control. This shows that *H. syriacus* had a more sustained and stronger effect on reducing erythema compared to the positive control (prednisolone) over the 5 week period. Increased melanin levels were observed in the 1% *H. syriacus*-treated group, while prednisolone treatment led to a reduction in melanin content (Figure 5G). Notably, the melanin levels in the *H. syriacus*-treated group were similar to those in the normal group, indicating a minimal effect on melanin production compared to the DNCB-treated control. The DNCB treatment led to a significant decrease in skin hydration. While prednisolone initially restored hydration, *H. syriacus* demonstrated superior efficacy in maintaining hydration after long-term treatment (Figure 5H). TEWL was significantly elevated in the DNCB group. However, both prednisolone and *H. syriacus* treatments reduced TEWL by 34.2% and 36.4%, respectively, indicating improved skin barrier function by the end of the study period (Figure 5I). The skin surface pH in the DNCB-treated group increased by 136.4%. Treatments with *H. syriacus* and prednisolone returned the pH to a slightly acidic level (6.34 and 6.41, respectively), similar to the normal group, indicating a normalization of the skin’s acidic barrier (Figure 5J).

### 2.4. Impact of Hibiscus syriacus Fatty Acid-Rich Fraction on Pro-Inflammatory Signaling

**Figure 6:**
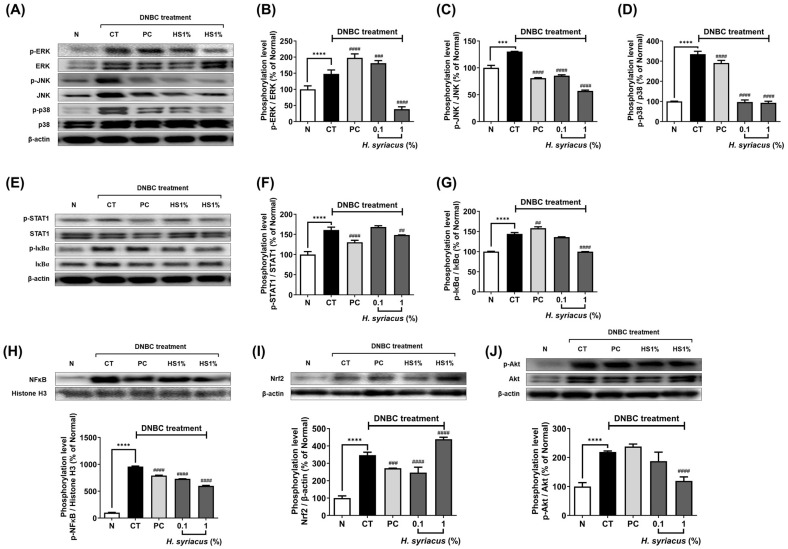
Effects of Hibiscus syriacus fatty acid-rich fraction on phosphorylation of inflammatory signaling proteins in DNCB-treated NC/Nga mice. Western blot and densitometric analyses were conducted to evaluate the phosphorylation levels of (**A**–**D**) MAPK proteins ((**A**) representative Western blot bands, (**B**) ERK, (**C**) JNK, and (**D**) p38) in dorsal skin tissues. (**E**–**G**) Phosphorylation of canonical inflammatory pathway proteins with (**E**) representative bands, (**F**) STAT1, and (**G**) IκBα. (**H**) NF-κB nuclear translocation with representative blot and quantitative graphs. (**I**) Nrf2 and (**J**) Phosphorylation of AKT with Western blot and corresponding densitometric analyses. N represents the normal group, CT indicates the DNCB-treated control group, and PC denotes the positive control group treated with 0.01% prednisolone. Data are presented as the mean ± SD; *** *p* < 0.001, **** *p* < 0.0001 vs. control group; ## *p* < 0.01, ### *p* < 0.001, #### *p* < 0.0001 vs. control group.

A Western blot analysis showed marked elevation in the phosphorylation of ERK, JNK, and p38 MAPKs in DNCB-treated NC/Nga mice, with increases of 147.8%, 130.3%, and 334.7%, respectively, when compared to the normal group (Figure 6A–D). Administration of *H. syriacus* (1%) led to a reduction in the activation of these proteins, lowering phosphorylation levels by 25.9%, 43.8%, and 27.9% for ERK, JNK, and p38, respectively, relative to the DNCB-treated control group. Prednisolone treatment also reduced the phosphorylation of JNK and p38 by 62.1% and 87.0%, respectively, but its effect on ERK was less pronounced (Figure 6B–D).

Phosphorylation of STAT1 and IκBα was significantly elevated by 161.1% and 143.8%, respectively, in the DNCB-treated group compared to the normal mice (Figure 6E–G). Treatment with *H. syriacus* significantly decreased the phosphorylation of these proteins, reducing STAT1 and IκBα levels by 80.9% and 69.3%, respectively, in a dose-dependent manner (Figure 6F–G). Furthermore, nuclear translocation of NF-κB was increased by an extraordinary 958.2% in DNCB-treated mice relative to the normal controls. However, the 1% *H. syriacus* diet reduced NF-κB nuclear expression by 62.6%, showing stronger efficacy than prednisolone, which exhibited a lesser reduction in NF-κB levels (Figure 6H).

AKT phosphorylation was markedly elevated by 219.2% in the DNCB-treated group compared to the normal group (Figure 6I). *H. syriacus* treatment led to a notable decrease in AKT phosphorylation by 54.4%, whereas the prednisolone diet showed a modest increase in AKT activation by 8.6% compared to the DNCB-treated control (Figure 6I).

## 3. Discussion

**Figure 7:**
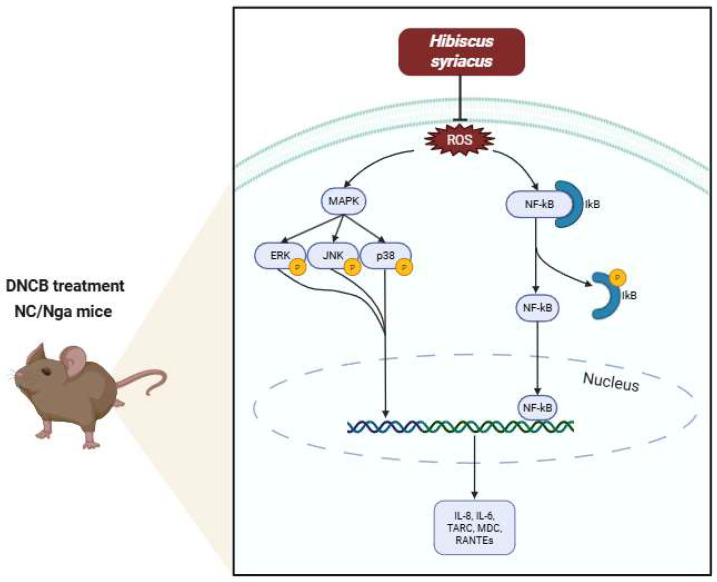
Schematic mechanism of *Hibiscus syriacus* fatty acid-rich fraction’s effect on atopic dermatitis.

This study provides compelling evidence that *Hibiscus syriacus* L. represents a novel, multi-target therapeutic strategy for AD that addresses both the inflammatory and barrier dysfunction components of the disease. Through integrated phytochemical characterization, computational modeling, and biological validation, these findings support *H. syriacus* as both a mechanistically validated and clinically promising alternative to conventional AD therapies that are limited by their single-target approach and adverse effect profiles (Figure 7). The GC-MS analysis identified palmitic acid (14.1%) and linoleic acid (10.3%) as the predominant fatty acids in the n-hexane fraction, with comparable relative abundances to those previously quantified by HPLC-DAD, despite methodological differences in detecting total methylated fatty acids (GC-MS) versus native free fatty acids (HPLC-DAD).

In terms of clinical advantages, *Hibiscus syriacus* offers distinct benefits over conventional and emerging therapies for AD, supporting its use as a safe, long-term option. Treated mice showed marked improvements in skin lesions, scratching behavior, TEWL, and hydration, along with reduced skin pH and serum IgE—factors that worsen pruritus (Figure 4). Although melanin levels were unaffected, erythema declined, with some observations possibly influenced by increased hair growth. Unlike corticosteroids such as prednisolone, which lose efficacy after 3–4 weeks and cause skin atrophy and rebound flares in 40% of users [19,20,21], *H. syriacus* sustained symptom control without adverse effects. Compared to tacrolimus, which frequently causes burning, stinging, pruritus, pain, and erythema [19,22], *H. syriacus* matched its TEWL-reducing effect while lowering the recurrence risk (Figure 4). Furthermore, unlike JAK inhibitors or biologics such as dupilumab that pose infection or injection-site risks [23,24], *H. syriacus* reduced IgE and inflammation comparably without immunosuppression. These findings highlight its promise for corticosteroid-sensitive or long-term AD management [5].

From a cytokine-driven skin inflammation perspective, the *Hibiscus syriacus* fatty acid-rich fraction exhibits broad yet selective anti-inflammatory activity against TNF-α- and IFN-γ-induced cytokine storms in keratinocytes. Unlike corticosteroids such as prednisolone, which globally suppress immune function and reduce lymphocyte activity by up to 70% [25], *H. syriacus* selectively downregulates pathogenic mediators while preserving skin barrier function. Specifically, it significantly suppressed IL-6 (by approximately 85.1%), TARC/CCL17 (by 73.6%), and MDC/CCL22 (by 71.4%) mRNA expression following cytokine stimulation. In contrast, corticosteroids reduce these markers but also downregulate antimicrobial peptides and structural proteins such as filaggrin, potentially compromising the skin’s defense [26,27,28]. Notably, palmitic acid, a major fatty acid component of *H. syriacus*, independently suppressed IL-6 (87.9%) and MDC/CCL22 (60.7%), surpassing tacrolimus in certain outcomes (Figure 3). Additionally, *H. syriacus* preserved the expression of barrier-related genes and showed no cytotoxicity up to 100 µg/mL. This suggests that unlike conventional treatments, which broadly inhibit immune signaling and risk rebound inflammation [5,29], *H. syriacus* offers a more targeted, non-immunosuppressive alternative for managing cytokine-driven skin inflammation. Additionally, *H. syriacus* achieves over 3-fold broader pathway coverage. This herb significantly suppresses NF-κB and STAT1, with experimental validation showing a 63% reduction in NF-κB p65 phosphorylation (Figure 6). This is a notable increase compared to the partial inhibition typically observed with corticosteroids, which generally suppress NF-κB activation by up to 50%, as reported in previous studies [30,31].

For clinical translation and future perspectives, the preclinical efficacy of *H. syriacus*, along with its favorable tolerability compared to corticosteroids, positions it as a promising candidate for AD management. While prednisolone treatment was associated with epidermal thinning and potential barrier disruption, *H. syriacus* preserved the skin architecture and improved hydration, TEWL, and the pH balance throughout the 5 week study. These benefits were achieved without systemic toxicity, weight loss, or signs of immunosuppression, common concerns in current AD therapies. Although this does not confirm the extract as entirely safe, it indicates a lower risk of treatment-related side effects at therapeutic doses (Figure 4).

Notably, PA and LA represent major constituents of the *H. syriacus* extract [16] and contribute to the extract’s biological activity, as both have been previously reported to exhibit anti-atopic dermatitis effects [12,13,16,32,33,34,35]. Additionally, our in vitro results support this view. PA significantly reduced reactive oxygen species (ROS) production (Figure 3D), and both PA and LA suppressed pro-inflammatory cytokine expression in TNF-α/IFN-γ-stimulated HaCaT cells (Figure 4A–I), consistent with their known anti-inflammatory roles in skin disorders. However, these fatty acids alone cannot account for the complete therapeutic effects, since all experimental groups received equivalent PA/LA doses through corn oil supplementation—yet only the *H. syriacus*-treated group demonstrated significant improvements. These findings suggest that synergistic interactions with co-extracted lipophilic compounds (phytosterols, tocopherols, and wax esters) likely enhance the biological activity, warranting future investigation through isobolographic or combination index analyses.

With oral bioavailability exceeding 90% (Table 1) and sustained therapeutic effects at low doses (1% dietary concentration), this natural extract holds the potential to revolutionize the management of AD through several key approaches. Targeted formulation strategies, such as the use of nanoemulsions, could lead to enhanced skin penetration, potentially increasing the efficacy by threefold compared to crude extracts [36]. Additionally, combination therapies with low-dose steroids are projected to reduce corticosteroid use by 50% [37]. Population-specific advantages further bolster its clinical promise.

Despite these encouraging findings, we acknowledge several limitations that merit further attention. First, the current model is based on acute DNCB-induced atopic dermatitis (AD), which may not fully replicate the chronic and relapsing nature of the condition in humans. Long-term safety data beyond the five-week study period are not yet available and will require further validation. One additional limitation is the use of a single concentration of the fatty acid–rich extract without a detailed dose–response assessment. This will be addressed in future studies using LC-MS/MS-based lipidomics combined with multi-dose experimental designs to better define concentration-dependent effects and contributions from specific lipid species. Although lymphocyte activity was not directly assessed, the reduction in serum IgE and dermal infiltration of mononuclear cells (Figure 5) suggests that *H. syriacus* extract may influence T cell-mediated immune responses involved in atopic dermatitis. Future studies should incorporate immunophenotyping approaches, such as flow cytometry [38] or in situ staining for CD4^+^, CD8^+^, and B220^+^ cells [39], to clarify its effects on lymphocyte subsets and functional activation [40].

## 4. Materials and Methods

### 4.1. Chemicals and Reagents

Dried flowers of *H. syriacus* were obtained from Bestherb (Seoul, Republic of Korea). Dulbecco’s modified Eagle’s medium (DMEM), fetal bovine serum (FBS), antibiotics, and trypsin-EDTA were purchased from Gibco-BRL (Grand Island, NY, USA). Lipopolysaccharide (LPS), recombinant human tumor necrosis factor-alpha (TNF-α), interferon-gamma (IFN-γ), dimethyl sulfoxide (DMSO), and 3-(4,5-dimethylthiazol-2-yl)-2,5-diphenyltetrazolium bromide (MTT) were purchased from Sigma-Aldrich (St. Louis, MO, USA). Enzyme-linked immunosorbent assay (ELISA) kits were procured from R&D Systems (Minneapolis, MN, USA). Primary and secondary antibodies were obtained from Santa Cruz Biotechnology (Dallas, TX, USA) and Cell Signaling Technology (Danvers, MA, USA). DCFH-DA was purchased from Thermo Fisher Scientific (Waltham, MA, USA). The Griess reagent system was obtained from Promega (Madison, WI, USA). TRIzol reagent was purchased from Invitrogen (Carlsbad, CA, USA). An ELISA reader was obtained from Molecular Devices (San Jose, CA, USA). Enhanced chemiluminescence (ECL) reagents were acquired from GE Healthcare (Chicago, IL, USA).

### 4.2. Preparation of Hibiscus syriacus Fatty Acid-Rich Fraction

Dried flowers of *H. syriacus* were collected from Bestherb (Seoul, Republic of Korea) and taxonomically identified by Professor Tae-Hoo Yi, Department of Oriental Medicine Biotechnology, Graduate School of Biotechnology, Kyung Hee University, Republic of Korea. A specimen voucher of *H. syriacus* has been deposited at Kyung Hee University Global Campus, assigned Voucher Specimen No. HYS2025-0456, located in Yongin, Gyeonggi-do, Republic of Korea. Dried *H. syriacus* flowers (4 kg) were extracted with hexane (1:10, *w*/*v*) at room temperature for 24 h using a Twist shaker (BioFree, Seoul, Republic of Korea). The process was repeated three times, and the fatty acid-rich fraction was filtered using Whatman filter paper No. 1 (Cytiva, Marlborough, MA, USA) and evaporated under vacuum conditions using a rotary evaporator (Heidolph Instruments, Schwabach, Germany) to achieve the *H. syriacus* flower fatty acid-rich fraction.

### 4.3. Gas Chromatography–Mass Spectrometry (GC-MS) Analysis

The chemical composition of the *H. syriacus* fatty acid-rich fraction was analyzed using an Agilent 6890 GC system (Agilent Technologies, Santa Clara, CA, USA) equipped with a CP-Wax column (50 m × 0.53 mm, 1.00 μm, Agilent Technologies). A 1 μL sample was injected at a flow rate of 6.0 mL/min. Fractionation was conducted using 0.5 N NaOH in methanol, BF3/methanol, and hexane (Sigma-Aldrich, St. Louis, MO, USA). This method converts both free and esterified fatty acids to methyl esters; therefore, the results represent the total fatty acid content. Quantification was performed using calibration curves constructed from authentic standards of palmitic acid and linoleic acid (Sigma-Aldrich, St. Louis, MO, USA).

### 4.4. Computational Docking

The key bioactive compounds of *H. syriacus* were selected for molecular docking studies. The 2D structures of these compounds were retrieved from the PubChem database and converted into 3D minimized structures using the LigPrep wizard in Schrödinger Maestro (v13.9). Epik was used to generate the ionization states of the compounds at the target pH of 7.0 ± 2.0, accounting for tautomer enumeration and protonation states relevant to biological conditions. A maximum of 32 stereoisomers per ligand were generated while retaining chiral specifications. The OPLS4 force field was employed for ligand minimization.

Protein structures were retrieved and prepared using the Protein Preparation Wizard in Schrödinger Maestro (v13.9). The preparation included assigning bond orders, adding hydrogen atoms, creating zero-order bonds to metal ions, creating disulfide bonds, converting selenomethionines to methionines, and removing water molecules within 5 Å of heteroatoms. The pH was set to 7.0 ± 2.0, and restrained minimization was performed using the OPLS4 force field with an RMSD convergence of 0.30 Å for heavy atoms.

#### 4.4.1. Identification of Active Sites of the Receptors

Active sites of proteins were identified using the Protein Preparation server to assess their druggability and define the key binding regions on the receptor surfaces. These sites are crucial for the binding and modulation of the ligands, which are essential for the therapeutic effects observed in inflammation and skin barrier restoration.

#### 4.4.2. Receptor Grid Generation

Receptor grid generation was performed in Schrödinger Maestro (v13.9) to define the binding sites for molecular docking. The van der Waals radius scaling factor was set to 1.0, with a partial charge cutoff of 0.25. The grid box was carefully positioned to cover the entire active site, ensuring comprehensive coverage for accurate docking simulations.

#### 4.4.3. Molecular Docking Simulation

Molecular docking simulations were carried out using the Glide Emodel method in Schrödinger Maestro (v13.9). This method is particularly suited for lipid compounds such as linoleic acid and palmitic acid, providing an accurate estimate of ligand–protein binding based on their lipidic nature. To account for non-cis/trans amide bonds, appropriate penalties were applied. The van der Waals scaling factor was set to 0.80, and the partial charge cutoff was 0.15. Glide Emodel providing more precise binding estimates for the lipidic compounds.

#### 4.4.4. ADME and QikProp Analyses

To evaluate the pharmacokinetic behavior of *Hibiscus syriacus* compounds, an ADME analysis was conducted using the ADME property prediction panel in Schrödinger Maestro (v13.9). The platform estimates the absorption potential, oral bioavailability, blood–brain barrier penetration, and metabolic stability based on structural features. Key parameters such as the oral absorption rate, predicted Caco-2 and MDCK cell permeability, and blood–brain barrier partitioning were analyzed to assess the suitability for systemic use in atopic dermatitis. The QikProp tool evaluates key pharmacokinetic parameters and helps predict the absorption, distribution, and metabolic properties of the compounds. The parameters considered include the molecular weight (acceptable range under 500), hydrogen bond donor count (acceptable range under 5), hydrogen bond acceptor count (acceptable range under 10), lipophilicity (LogP, acceptable range under 5), and molar refractivity (acceptable range 40—130). Lipinski’s rule of five was used to evaluate the drug-likeness of the compounds. QikProp also provided information on the bioavailability and toxicity profiles of the compounds, which are critical for determining their potential as therapeutic agents.

### 4.5. Cell Culture and Treatments

Murine macrophage RAW 264.7 and human keratinocyte HaCaT cells were obtained from ATCC (Manassas, VA, USA) and maintained in DMEM with 10% FBS and 1% antibiotics at 37 °C in a 5% CO2 atmosphere. For cultivation, HaCaT cells were grown in 100 mm dishes, while RAW 264.7 cells were maintained in T-75 flasks. To prepare for experiments, cells were plated in 6-well plates (HaCaT, 1.0 × 105 cells) and 96-well plates (RAW 264.7, 1.0 × 105 cells) and allowed to grow until they reached 80% confluence. Cells were treated with *H. syriacus* (1, 10, and 100 μg/mL) following stimulation with 1 μg/mL of LPS (RAW 264.7) or 10 ng/mL of TNF-α and IFN-γ (HaCaT). Concentrations for isolated compounds were selected based on cytotoxicity screening and fractional abundance in the extract, aligning with physiologically relevant doses from previous studies [13,41].

### 4.6. Measurement of Nitric Oxide (NO) Production

Nitric oxide (NO) production in RAW 264.7 cells was assessed using the Griess reagent system (Promega, Fitchburg, WI, USA). Following treatment, 100 μL of supernatant was transferred to a new 96-well plate. Next, 50 μL of sulfanilamide solution and 50 μL of *N*-(1-naphthyl)ethylenediamine dihydrochloride solution were added to each well. The mixture was then incubated for 10 min at room temperature, and absorbance was measured at 595 nm using a Molecular Devices FilterMax F5 ELISA reader (San Francisco, CA, USA). Dexamethasone (10 μg/mL) was used as a positive control for LPS-activated RAW 264.7 cells in a 96-well plate.

### 4.7. Measurement of Reactive Oxygen Species (ROS)

After a 24 h sample treatment and sensitization, cells were incubated with 30 µM of 2′,7′-dichlorofluorescein diacetate (DCFH-DA; Sigma-Aldrich, St. Louis, MO, USA) for 30 minutes at 37 °C in the dark. Following incubation, cells were washed with PBS and harvested using trypsin-EDTA. The intracellular ROS levels were quantified using a BD Accuri C6 flow cytometer (BD Biosciences, San Jose, CA, USA). Data were subsequently analyzed with FCS Express 6 Plus Research Edition (2017, De Novo Software, Pasadena, CA, USA).

### 4.8. Cell Viability Assay

Cell viability was determined using the MTT assay. At the end of the treatment period, MTT reagent was added to the cells to achieve a final concentration of 0.1 mg/mL. The cells were then incubated for 2 h at 37 °C in a CO_2_ incubator. After incubation, the medium was removed and 800 μL of dimethyl sulfoxide (DMSO) was added to dissolve the formazan crystals. Absorbance was measured at 595 nm using a Molecular Devices FilterMax F5 ELISA reader (San Francisco, CA, USA).

### 4.9. Reverse Transcription Polymerase Chain Reaction (RT-PCR)

TRIzol reagent (Invitrogen, Grand Island, NY, USA) was used to extract total RNA from HaCaT cells. RNA concentrations were measured, and 4 μg of total RNA was utilized for reverse transcription. Reverse transcription was performed using 200 units of reverse transcriptase and 0.5 μg/μL of oligo-(dT)15 primers (Bioneer, Daejeon, Republic of Korea). A PCR premix (Bioneer, Daejeon, Republic of Korea) was used for the amplification process. TARC/CCL17, MDC/CCL22, RANTES, IL-6, and IL-8 primers were used as outlined in Table 3. A Veriti Thermal Cycler (Applied Biosystems, Foster City, CA, USA) was used for the PCR reaction. The 2.0% agarose gel electrophoresis was performed to separate the PCR products, and ethidium bromide was used for visualization.

### 4.10. Enzyme-Linked Immunosorbent Assay (ELISA)

Cytokine and chemokine secretion levels were measured using commercially available ELISA kits, specifically designed for Human CCL17/TARC, Human CCL22/MDC, and Human CCL5/RANTES (Quantikine ELISA Kits, R&D Systems, Inc., Minneapolis, MN, USA). Following a 24 h sensitization and treatment period, 1 mL of cell culture supernatant was collected. The concentrations of TARC/CCL17, RANTES/CCL5, and MDC/CCL22 were then quantified according to the instructions provided by the kit manufacturer.

### 4.11. Western Blot

Skin tissues and cells were collected, and the separation of proteins into nuclear and cytoplasmic fractions was conducted using a commercial extraction kit (NE-PER Nuclear and Cytoplasmic Extraction Reagents; Pierce). Protein concentrations were measured using Bradford reagent (Bio-Rad, Hercules, CA, USA), and homogenized cell and skin lysates were prepared to ensure equivalent protein amounts. Using 10–15% sodium dodecyl sulfate-polyacrylamide gel electrophoresis (SDS-PAGE), we separated the proteins and transferred them to a nitrocellulose membrane (Amersham Pharmacia Biotech, Buckinghamshire, UK). Blocking of non-specific binding sites was performed with 5% bovine serum albumin in TBST (50 mM Tris-HCl, pH 7.5, 150 mM NaCl, and 0.1% Tween 20) for 1 h at room temperature. The membranes incubated overnight at 4 °C with primary antibodies, washed three times with TBST, and then treated with secondary antibodies (Santa Cruz Biotechnology Inc., Dallas, Texas, CA, USA) for 1 h at room temperature. Chemiluminescence detection was carried out using ECL reagent (GE Healthcare Life Sciences, Marlborough, Massachusetts, PA, USA), and protein quantification was performed with UVI-1D software (UVITEC, Cambridge, Warwickshire, UK).

### 4.12. Animals and Experimental Design

Six-week-old female NC/Nga mice (20.0–20.3 g; *n* = 25) were sourced from Central Lab Animals, Inc. (Seoul, Republic of Korea). The mice were randomly assigned into five groups, with two mice per cage, and housed under controlled conditions of 22 ± 1 °C, 60 ± 5% humidity and a 12 h light/dark cycle. The experimental protocol (KHUASP (SE)-17-014) was approved by Kyung Hee University’s Institutional Animal Care and Use Committee. Following a one-week adaptation period, the 25 mice were divided into five groups as follows: (a) normal (control diet only), (b) control (DNCB treatment with control diet), (c) 0.01% prednisolone (DNCB treatment with diet containing 0.01% prednisolone), (d) 0.1% *H. syriacus* (DNCB treatment with diet containing 0.1% *H. syriacus*), and (e) 1% *H. syriacus* (DNCB treatment with diet containing 1% *H. syriacus*). DNCB was applied daily during the first week to induce atopic dermatitis-like lesions on the dorsal skin. Throughout the experimental period, the mice received 0.2% DNCB topical treatment three times per week for four weeks. Additionally, AIN93G feed mixed with *H. syriacus* or prednisolone was provided ad libitum according to the dietary composition (Table 4). The diet composition used in this study was based on the standardized AIN-93G formulation with modifications widely employed in skin inflammation and immune response models to ensure consistent nutrition and minimize dietary variability [42,43].

### 4.13. Evaluation of Atopic Dermatitis-like Symptoms

The severity of atopic dermatitis-like lesions on the dorsal skin was observed using a video scope probe, and photographs were taken weekly at the center of the dorsal skin throughout the experimental period. The severity was assessed based on criteria such as erythema, crusts, excoriation, and lichenification.

During the experimental period, physical alterations in the dorsal skin of the experimental animals were measured using the Dermalab^®^ combo skin measurement instrument (Coretex Technology, Aalborg, Denmark). Various skin parameters, including high-frequency ultrasound, TEWL, hydration, erythema, melanin content, and pH, were measured using the appropriate probes. All data were analyzed using the Dermalab skinlab software (Coretex Technology, Aalborg, Denmark).

The total number of scratching behaviors was recorded weekly throughout the experimental period. Each group of mice was placed in a transparent plastic container, and their behavior was videotaped for 15 min. The frequency of scratching behaviors on the nose, ears, and dorsal skin was recorded during the video recording. Scratching was defined as the raising of the hind paw followed by repeated scratching movements.

At the end of the experimental period, blood samples were collected from the mice, and the samples were centrifuged at 14,000× *g* for 20 min at 4 °C. The supernatants were then collected, and the total serum IgE levels were measured using a mouse IgE enzyme-linked immunosorbent assay kit (BD Bioscience, CA, USA), according to the manufacturer’s instructions.

The dorsal skin from the experimental animals was fixed in paraformaldehyde for 24 h and then embedded in paraffin blocks. The blocks were sliced into 4-µm-thick sections for hematoxylin and eosin (H&E) staining. The process included deparaffinization, hydration, H&E staining, and dehydration to observe histopathological changes in the dorsal skin lesions of NC/Nga mice. The stained skin samples were examined under a microscope.

### 4.14. Statistical Analysis

Data were analyzed using GraphPad Prism 10 (GraphPad Software, San Diego, CA, USA) and presented as the mean ± SD. Statistical significance was determined using one-way and two-way ANOVAs followed by Duncan’s test (*p <* 0.05, *p <* 0.01, *p <* 0.001, and *p <* 0.0001).

## 5. Conclusions

Our study identifies *H. syriacus* as preclinically promising for use as a functional food or oral medicine option for AD, addressing the critical need for safer, multi-targeted alternatives to topical corticosteroids. Through integrated molecular docking and in vivo validation, its active constituents, linoleic and palmitic acids, were shown to target key nodes in the NF-κB inflammatory pathway with high efficacy. The extract significantly attenuated inflammatory mediators and MAPK/NF-κB signaling, while in vivo treatment in a DNCB-induced AD mouse model achieved corticosteroid-comparable anti-inflammatory outcomes without inducing skin atrophy. By simultaneously resolving the inflammation and restoring the skin barrier function, *H. syriacus* presents a compelling therapeutic strategy for AD and a promising platform for the further development of safe, multi-target botanical treatments.

## Figures and Tables

**Table 1:** QikProp-predicted physicochemical and ADME properties of linoleic acid and palmitic acid.

Category	Linoleic Acid	Palmitic Acid
Molecular weight	280.45	256.43
LogP (QPlogPo/w)	5.291 → highly lipophilic	5.251 → highly lipophilic
LogS (QPlogS)	−4.635 → poorly soluble	−5.497 → very poorly soluble
Human oral absorption	3 (high)/87.5%	3 (high)/87.2%
# of H-bond donors	1	1
# of H-bond acceptors	2	2
Dipole moment	6.52	6.83
Polar surface area	37.5 Å^2^	0.0 Å^2^ (due to lack of polar groups)
CNS activity	−2 (inactive)	−2 (inactive)
QPPCaco (permeability)	239.37 nm/s → good	235.46 nm/s → good
# Rule of 5 violations	1	1
# of rotatable bonds	14	14
Solvent accessible SA	624.03 Å^2^	670.70 Å^2^

LogP (QPlogPo/w): octanol/water partition coefficient; LogS (QPlogS): aqueous solubility; CNS: central nervous system activity; QPPCaco: Caco-2 cell permeability; H-bond: hydrogen bond; SA: surface area; rule of 5: Lipinski’s rule for drug-likeness.

**Table 2:** Binding affinity prediction of linoleic and palmitic acids using Glide Emodel.

Protein	Linoleic Acid	Palmitic Acid
NF-κB	−26.656	−17.991
IKKβ	−22.503	−22.781
STAT1	−23.324	−19.525
AKT	−35.18	−22.423
JNK	−15.747	−12.312
ERK	−26.669	−18.114
p38	−30.551	−24.713

**Table 3:** List of primers for the RT-PCR analysis.

Gene	Primer Type	Sequence (5′-3′)
GAPDH	Forward	5′-ACCACAGTCCATGCCATCAC-3′
	Reverse	5′-CCACCACCCTGTTGCTGTAC-3′
IL-6	Forward	5′-CTCCTTCTCCACAAGCGCC-3′
	Reverse	5′-GCCGAAGAGCCCTCAGGC-3′
IL-8/CXCL8	Forward	5′-TCAGTGCATAAAGACATACTCC-3′
	Reverse	5′-TGGCATCTTCACTGATTCTTG-3′
TARC/CCL17	Forward	5′-ATGGCCCCACTGAAGATGCT-3′
	Reverse	5′-TGAACACCAACGGTGGAGGT-3′
RANTES/CCL5	Forward	5′-CCCCGTGCCGAGCACATCAAGGAGTATTT-3′
	Reverse	5′-CGTCCAGCCTGGGGAAGGTTTTTGTA-3′
MDC/CCL22	Forward	5′-AGGACAGAGCATGGCTCGCCTACAGA-3′
	Reverse	5′-AATGGCAGGGAGGTAGGGCTCCTGA-3′

**Table 4:** Dietary composition.

**Compositions**	**Normal (*n* = 5)**	**Control (*n* = 5)**	**Prednisolone 0.01% (*n* = 5)**	***H. syriacus* 0.1% (*n* = 5)**	***H. syriacus* 1% (*n* = 5)**
Casein	230	230	230	230	230
L-cystine	3	3	3	3	3
Corn oil	100	100	100	100	100
Cellulose	50	50	50	50	50
Vitamin mix	10	10	10	10	10
Mineral mix	35	35	35	35	35
Sucrose	200	200	200	200	200
Corn starch	372	372	372	371	362
*H. syriacus*	−	−	−	1	10
DNCB	−	+	+	+	+

“+” denotes the presence (diet added); “−” denotes the absence (diet not added).

## Data Availability

The data presented in this study are available in this paper.

## Abbreviations

The following abbreviations are used in this manuscript:

AD	Atopic Dermatitis
NO	Nitric Oxide
ROS	Reactive Oxygen Species
TNF-α	Tumor Necrosis Factor-Alpha
IFN-γ	Interferon-Gamma
TARC	Thymus and Activation-Regulated Chemokine (CCL17)
MDC	Macrophage-Derived Chemokine (CCL22)
RANTES	Regulated on Activation, Normal T Cell Expressed and Secreted (CCL5)
IL-6	Interleukin-6
IL-8	Interleukin-8
MAPK	Mitogen-Activated Protein Kinase
ERK	Extracellular Signal-Regulated Kinase
JNK	c-Jun N-Terminal Kinase
p38	p38 Mitogen-Activated Protein Kinase
NF-κB	Nuclear Factor Kappa-Light-Chain-Enhancer of Activated B Cells
IKKβ	Inhibitor of Nuclear Factor Kappa-B Kinase Subunit Beta
STAT1	Signal Transducer and Activator of Transcription 1
AKT	Protein Kinase B
DNCB	2,4-Dinitrochlorobenzene
TEWL	Transepidermal Water Loss
